# Factors associated with deaths due to visceral leishmaniasis among children at a hospital in the southwest of Maranhão state: a cross-sectional study, Imperatriz, 2010-2021

**DOI:** 10.1590/S2237-96222025v34e20240738.en

**Published:** 2025-09-29

**Authors:** Marcela de Maria Pereira Teixeira, Daniel Ferreira dos Santos, Ezequiel Almeida Barros, Leonardo Hunaldo dos Santos, Laise Sousa Siqueira, Livia Maia Pascoal, Ana Cristina Pereira de Jesus Costa, Marcelino Santos

**Affiliations:** 1 Universidade Federal do Maranhão, Centro de Ciências de Imperatriz, Imperatriz, MA, Brazil

**Keywords:** Leishmaniosis, Visceral, Clinical Evolution, Child, Public Health Surveillance, Cross-Sectional Studies, Leishmaniosis Visceral, Evolución Clínica, Niño, Vigilancia en Salud Pública, Estudios Transversales

## Abstract

**Objectives:**

To describe the clinical and epidemiological characteristics and analyze factors associated with deaths due to visceral leishmaniasis among children at a hospital in the southwest of Maranhão state.

**Methods:**

This was a cross-sectional study conducted in Imperatriz, Maranhão, based on case reporting forms held on the Notifiable Health Conditions Information System provided by the Regional Epidemiological Surveillance and Disease Control Center. The study included all records of visceral leishmaniasis cases in children admitted to a pediatric referral hospital in Imperatriz between 2010 and 2021. Data were analyzed using descriptive statistics, determining absolute and relative values of the variables investigated. Odds ratios (OR) and 95% confidence intervals (95%CI) were calculated using univariate and multivariate regression.

**Results:**

A total of 404 cases of the disease were recorded, of which 43 resulted in death. Of these, the majority were new cases (95.4%), females (53.5%), of mixed race/skin color (65.1%), and resident in the urban zone (60.5%) who presented clinical manifestations such as enlarged spleen (88.4%), skin pallor (86.0%), weight loss (69.8%), edema (60.5%), fever (95.4%), and enlarged liver (51.2%). In the final regression model, living in the rural zone was considered a risk factor (OR 2.96; 95%CI 1.35; 6.50), while being between 1 and 4 years old was a protective factor (OR 0.23; 95%CI 0.11; 0.50) against death from the disease.

**Conclusion:**

The findings highlighted the need for targeted strategies to improve early diagnosis and management of visceral leishmaniasis, especially in rural areas.

## Resumen

**Objetivos:**

Describir las características clínicas y epidemiológicas y analizar los factores asociados a las muertes por leishmaniasis visceral en niños en un hospital del suroeste del estado de Maranhão.

**Métodos:**

Estudio transversal realizado en Imperatriz, Maranhão, basado en los formularios de notificación de casos del Sistema de Información de Enfermedades de Notificación Obligatoria, proporcionados por el Centro de Vigilancia Epidemiológica y Control de Enfermedades de la unidad regional. El estudio incluyó todos los registros de casos de leishmaniasis visceral en niños ingresados en un hospital pediátrico de referencia en Imperatriz entre 2010 y 2021. Los datos se analizaron mediante estadística descriptiva, determinando los valores absolutos y relativos de las variables investigadas. Se calcularon las oportunidades relativas (*odds ratios* - OR) y los intervalos de confianza del 95% (IC95%) mediante regresión univariante y multivariante.

**Resultados:**

Se registraron 404 casos de la enfermedad, de los cuales 43 resultaron en fallecimiento. De estos, la mayoría fueron casos nuevos (95,4%), niñas (53,5%), raza/color de piel pardo (65,1%), residentes en la zona urbana (60,5%) que presentaron manifestaciones clínicas como esplenomegalia (88,4%), palidez de la piel (86,0%), pérdida de peso (69,8%), presencia de edemas (60,5%), fiebre (95,4%) y hepatomegalia (51,2%). En el modelo de regresión final, vivir en la zona rural se consideró un factor de riesgo (OR 2,96; IC95% 1,35; 6,50), y la edad entre 1 y 4 años fue un factor protector (OR 0,23; IC 95% 0,11; 0,50) para fallecer por la enfermedad.

**Conclusión:**

Los hallazgos destacaron la necesidad de estrategias específicas para mejorar el diagnóstico temprano y el tratamiento de la leishmaniasis visceral, especialmente en las zonas rurales.

**Ethical aspects**

This research respected ethical principles, having obtained the following approval data:

Research Ethics Committee: Universidade Federal do Maranhão

Opinion number: 5,656,905

Approval date: 21/9/2022

Certificate of Submission for Ethical Appraisal: 57683422.1.0000.5086

Informed Consent Form: Waived.

## Introduction

Visceral leishmaniasis is a chronic tropical disease caused by the protozoan *Leishmania chagasi*, a member of the Trypanosomatidae family (1). In Brazil, transmission occurs through the bite of an infected female sand fly, Lutzomyia longipalpis, with domestic dogs being the main reservoir. The incubation period ranges from 10 to 14 days, and symptoms may appear between two and eight months after infection. Clinical manifestations include irregular episodes of fever, weight loss, weakness, enlarged spleen and liver, lymphadenopathy, and anemia (2). 

Global data from 2023 indicated that visceral leishmaniasis is endemic in 83 countries, and an estimated 50,000 to 90,000 new cases emerge each year. In 2018, more than 90.0% of new cases occurred in India, Bangladesh, Brazil and East Africa (3). 

According to the Pan American Health Organization, the Americas face significant challenges in controlling the disease due to its high incidence and wide geographic distribution. Brazil leads the continent in terms of cases, recording 7,928 between 2018 and 2020, followed by Venezuela, Paraguay, Colombia, Argentina, Bolivia and Uruguay (4).

In 2018, Brazil accounted for approximately 96.0% of visceral leishmaniasis cases in the Americas, and Northeast Brazil was the most affected region of the country (5). In 2020, of the 165 deaths recorded in Brazil, 107 occurred in the Northeast, and Maranhão was the state with the highest number of deaths among the country’s federative units, having 42 of these 165 cases (6-8).

If left untreated, Visceral leishmaniasis has a high case fatality ratio. Approximately 90% of cases can result in death due to systemic involvement caused by the presence of parasites in internal organs such as bone marrow, spleen, and liver. The disease primarily affects vulnerable populations, such as children under 5 years of age, the elderly, and patients with comorbidities and other immunosuppressive conditions, such as human immunodeficiency virus infection/acquired immunodeficiency syndrome and malnutrition.

In 2020, of 162 reported cases of visceral leishmaniasis deaths, 16.0% occurred among children under 5 years of age (4). In that same year, the disease’s case fatality ratio reached 9.5%, the highest in the preceding 10 years, with a worrying rate among children under 1 year of age, which reached 14.0% (8). Children form the population most affected by visceral leishmaniasis (9-12).

In-depth understanding of the epidemiology and progression of visceral leishmaniasis in pediatric populations is essential for developing effective prevention and control strategies. Despite advances in scientific research over the years, the specific factors that contribute to visceral leishmaniasis deaths among children remain unknown. It is assumed that the immunological vulnerability of these individuals, combined with their greater propensity for malnutrition, may worsen the clinical picture of the disease, increasing the risk of fatal outcomes (7,13). Given this knowledge gap, it is imperative to conduct studies that broaden the epidemiological understanding of visceral leishmaniasis in different endemic settings.

This study aimed to describe the clinical and epidemiological characteristics and analyze factors associated with visceral leishmaniasis deaths among children admitted to a hospital service in Imperatriz, a city in the state of Maranhão.

## Methods

### Design 

This was a cross-sectional study that investigated clinical and epidemiological factors associated with death due to leishmaniasis among children between 2010 and 2021 admitted to a hospital in Imperatriz.

### Setting

The study data were collected from the Notifiable Health Conditions Information System and provided by the Imperatriz Regional Epidemiological Surveillance and Disease Control Center. The data included records of visceral leishmaniasis in the 0-12 age group from a hospital in southwestern Maranhão, located in Imperatriz.

Imperatriz is the second-largest city in Maranhão and is part of the state’s southern health macro-region, along with municipalities such as Balsas, Açailândia and Barra do Corda. The Imperatriz Municipal Children’s Hospital is the only public pediatric referral center in the region, offering emergency services, surgeries, pediatric clinical care and an intensive care unit.

### Participants

All cases of deaths from visceral leishmaniasis recorded in children under 12 years of age admitted to the hospital between 2010 and 2021 were included in the study. However, it should be noted that, despite the aforementioned inclusion factor, deaths were only found for children aged 0 to 4 years.

### Variables

The variables analyzed included type of hospital admission, sex, age group, race/skin color, zone of residence, clinical manifestations (such as enlarged spleen, pallor, weight loss, edema, fever, jaundice, and enlarged liver), coinfection with human immunodeficiency virus, diagnosis methods, and diagnosis confirmation criteria.

### Data source and measurement 

Data collection was performed using visceral leishmaniasis reporting forms held on the Notifiable Health Conditions Information System. These data were provided by the Imperatriz Regional Epidemiological Surveillance and Disease Control Center, part of the Maranhão State Health Department. The variables were duly coded and recorded in accordance with the system’s standards, thus ensuring uniformity in data measurement.

### Bias control

Measures were adopted to minimize bias, including exclusion of unknown or incomplete data and verification of multicollinearity between variables, in order to ensure that the statistical analysis reflected the reality of the cases assessed.

### Sample size

The analysis included all available records of visceral leishmaniasis deaths among children under 12 years of age from 2010 to 2021. The sample comprised 43 cases of visceral leishmaniasis deaths during the period investigated.

### Data quality evaluation 

The collected data were reviewed to identify and correct possible inconsistencies or errors. Incomplete or unknown data were excluded to ensure the accuracy of the analysis. There was no multicollinearity between the independent variables evaluated. On this occasion, unknown data were excluded. All tests were performed using the Statistical Package for the Social Sciences (IBM SPSS) Statistics software with a 5% significance level (15).

### Statistical methods

The initial analysis was performed using univariate logistic regression (unadjusted) to verify associations between the variables and the outcome of death due to visceral leishmaniasis, taking a p-value<0.20 for selection. Significant variables were subsequently analyzed using multivariate logistic regression (adjusted) to estimate the odds ratios (OR) with 95% confidence intervals (95%CI), maintaining the 5% significance level (14).

## Results

Between 2010 and 2021, 404 cases of visceral leishmaniasis were confirmed in children under 12 years of age, of which 43 (7.7%) resulted in death. The majority of deaths were new cases (41; 95.4%), female (23; 53.5%), of mixed race/skin color (28; 65.1%), and resident in the urban zone (26; 60.5%). The main clinical manifestations included enlarged spleen (38; 88.4%), pallor (37; 86.0%), weight loss (30; 69.8%), edema (26; 60.5%), fever (41; 95.4%), absence of jaundice (24; 55.8%), and enlarged liver (22; 51.2%). Low occurrence of visceral leishmaniasis/human immunodeficiency virus coinfection (37; 86.0%) among the reported deaths (Table 1) stood out.

**Table 1:** Case number (n) and percentage (%) distribution according to clinical-epidemiological and laboratory characteristics of visceral leishmaniasis among children hospitalized in a pediatrics service in the southwest region of the state of Maranhão between 2010 and 2021. Imperatriz, 2025 (n=43)

Variables	n (%)
**Type of admission**	
New case	41 (95.4)
Recurrence	1 (2.3)
Unknown	1 (2.3)
Sex	
Male	20 (46.5)
Female	23 (53.5)
**Age group** (years)	
<1	20 (46.5)
1-4	21 (48.8)
Unknown	2 (4.7)
**Race/skin color**	
White	6 (14.0)
Mixed race	28 (65.1)
Indigenous	7 (16.2)
Unknown	2 (4.7)
**Zone of residence**	
Urbana	26 (60.5)
Rural	16 (37.2)
Peri-urban	1 (2.3)
**Enlarged spleen**	
Yes	38 (88.4)
No	3 (6.9)
Unknown	2 (4.7)
Pallor	
Yes	37 (86.0)
No	5 (11.7)
Unknown	1 (2.3)
**Weight loss**	
Yes	30 (69.8)
No	12 (27.9)
Unknown	1 (2.3)
Edema	
Yes	26 (60.5)
No	16 (37.2)
Unknown	1 (2.3)
Fever	
Yes	41 (95.4)
No	1 (2.3)
Unknown	1 (2.3)
Jaundice	
With jaundice	16 (37.2)
Without jaundice	24 (55.8)
Unknown	3 (7.0)
**Enlarged liver**	
Yes	22 (51.2)
No	18 (41.8)
Unknown	3 (7.0)
**Human immunodeficiency virus**	
Yes	1 (2.3)
No	37 (86.0)
Unknown	5 (11.7)
**Parasite diagnosis**	
Positive	7 (16.3)
Negative	1 (2.3)
Unknown	35 (81.4)
**Immunologic diagnosis**	
Positive	12 (27.9)
Negative	7 (16.3)
Unknown	24 (55.8)
**Confirmation criterion**	
Laboratory	25 (58.1)
Clinical-epidemiological	18 (41.9)

Regarding the confirmation criterion, the laboratory criterion (25; 58.1%) was the most evident. With regard to diagnosis of the disease, the parasite diagnosis variable (35; 81.4%) and the immunologic diagnosis variable (24; 55.8%) presented inconsistent data and consequently a higher frequency of unknown data (Table 1).

The crude analysis of the variables that showed significant association with death from visceral leishmaniasis identified the following risk factors: living in the rural zone (OR 2.19; 95%CI 1.10; 4.34), no weight loss (OR 1.68; 95%CI 1.02; 3.50), edema (OR 1.93; 95%CI 1.09; 3.74), jaundice (OR 2.13; 95%CI 1.07; 4.24), and clinical-epidemiological confirmation criterion (OR 1.62; 95%CI 1.09; 3.13) (Table 2). The 1 to 4 years age variable (OR 0.29; 95%CI 0.14; 0.56) proved to be a protective factor as it presented a p-value<0.001.

**Table 2:** Crude and adjusted odds ratios (OR) and 95% confidence intervals (95%CI) of deaths due to visceral leishmaniosis among children hospitalized in a pediatric service in the southwest region of the state of Maranhão between 2010 and 2021, according to the study variables. Imperatriz, 2025 (n=404)

Variables	Crude OR (95%CI)	p-value	Adjusted OR (95%CI)	p-value
**Type of admission**		0.380		-
New case	1.00			
Recurrence	0.40 (0.52; 3.09)		-	
Sex		0.300		-
Male	1.00			
Female	1.40 (0.74; 2.67)		-	
**Age group** (years)		<0.001		<0.001
<1	1.00			
1-4	0.29 (0.14; 0.56)		0.23 (0.11; 0.50)	
**Race/skin color**		0.570		-
Mixed race	1.26 (0.57; 2.79)		-	
Not mixed race	1.00			
**Zone of residence**		0.030		0.010
Urbana	1.00			
Rural	2.19 (1.10; 4.34)		2.96 (1.35; 6.50)	
Peri-urban			0.00	1
**Enlarged spleen**		0.630		-
Yes	1.36 (0.39; 4.69)		-	
No	1.00			
Pallor		0.700		-
Yes	1.00			
No	1.22 (0.44; 3.36)		-	
**Weight loss**		0.160		0.290
Yes	1.00			
No	1.68 (1.02; 3.50)		0.64 (0.28; 1.48)	
Edema		0.050		0.460
Yes	1.93 (1.09; 3.74)		1.33 (0.63; 2.86)	
No	1.00			
Fever		0.780		-
Yes	1.35 (0.17; 10.95)		-	
No	1.00			
Jaundice		0.030		0.180
Yes	2.13 (1.07; 4.24)		1.73 (0.78; 3.80)	
No	1.00			
**Enlarged liver**		0.990		-
Yes	1.00			
No	1.05 (0.52; 1.96)		-	
**Human immunodeficiency virus**		0.990		-
Yes	1.00			
No	1.02 (0.12; 8.50)		-	
**Parasite diagnosis**		0.910		-
Positive	0.14 (0.12; 10.94)		-	
Negative	1.00			
**Immunologic diagnosis**		0.520		-
Positive	1.00			
Negative	1.39 (0.51; 3.77)		-	
**Confirmation criterion**		0.150		0.200
Laboratory	1.00			
Clinical-epidemiological	1.62 (1.09; 3.13)		1.65 (0.76; 3.60)	

In the adjusted analysis, the living in the rural zone variable was shown to be a risk factor for death from visceral leishmaniasis was (OR 2.96; 95%CI 1.35; 6.50). The 1 to 4 years age variable (OR 0.23; 95%CI 0.11; 0.50) was shown to be a protective factor (Table 2).

## Discussion

In all, 404 cases of visceral leishmaniasis were recorded in the age group indicated, 43 of which resulted in death. Of these, the majority were new cases, female, of mixed race/skin color, and resided in the urban zone who presented clinical manifestations such as enlarged spleen, skin pallor, weight loss, edema, fever and enlarged liver. In the final regression model, living in the rural zone was considered a risk factor, while being between 1 and 4 years old was a protective factor for death from the disease.

A limitation of the study lies in the number of unknown data, which highlights variations in the quality of data entry and restricts the variables that could be explored. This limitation can generate biases in data interpretation and even indicate underreporting. Therefore, it is important to improve the quality of records, especially in the completion of case reports, as this information is fundamental for efficient healthcare planning and management. Another factor worthy of note is that, although we analyzed the 0-12 age group for visceral leishmaniasis dealths, we only found deaths among children up to 4 years of age.

When analyzing the clinical and epidemiological characteristics, the study found that the new case admission type variable presented a high percentage related to death due to the condition. In the state of Mato Grosso, a study found that the 0-4 years age group accounted for 24.2% of deaths, with a rate of 96.2% among new cases in 2022 (16). In the state of Pará, children constituted the particularly susceptible population, and, in 2021, the highest percentage of deaths (83.3%) occurred among cases of primary infection (13).

It was also found that female patients had a higher relative frequency of deaths compared to males. This diverged in relation to Brazilian scenarios, such as the states of Tocantins, Pará, Sergipe, Mato Grosso and Piauí, where males were the most affected (9,17-20). The fact that males are the most affected in terms of illness and death is not yet fully understood.

Due to the scarcity of studies providing evidence on this, one hypothesis is that the same physiological causes attributed to men in adulthood, related to the effects of sex hormones on the immune system, making them more susceptible to Leishmania, added to the fact that early childhood is the phase in which individuals have an immature immune system (21-23).

Deaths due to visceral leishmaniasis occurred more frequently in individuals of mixed race/skin color, consistent with data from Piauí in 2022, where mixed-race patients accounted for 100.0% of deaths (24). This significant percentage can be explained by the fact that visceral leishmaniasis is a neglected disease, predominantly affecting individuals in unfavorable socioeconomic conditions, a context in which the majority of the non-White population lives (20,23).

The highest incidence of deaths occurred in individuals residing in the urban zone, as was also found in Pará in 2021 (19). It is suggested that these findings are related to factors arising from the limitations faced by local health services, as well as a lack of knowledge about appropriate management of patients with visceral leishmaniasis. This includes late identification of the need to send serious cases to referral centers with greater technological capacity, which could contribute to reducing the case fatality ratio of the disease (25).

The results revealed the predominance of classic clinical manifestations of visceral leishmaniasis among pediatric patients, reinforcing the systemic and severe nature of the disease. Enlarged spleen and liver were common findings, consistent with the pathogenesis of visceral leishmaniasis, which frequently causes splenomegaly and hepatomegaly due to parasitic invasion of the lymphoid and liver organs. Fever and pallor stood out as important markers of infection and anemia, respectively, corroborating the literature that associates these symptoms with malnutrition and immunocompromised patients (9,20,26).

The high rate of weight loss and edema further suggests the severe nutritional impact and metabolic impairment caused by the disease, exacerbated by unfavorable socioeconomic conditions, which contribute to the debilitating clinical picture of these children. These findings highlight the urgent need for early interventions to prevent fatal complications and improve the clinical management of pediatric cases (9,20,26).

This study highlighted the absence of visceral leishmaniasis/human immunodeficiency virus coinfection, a finding that contrasts with other global scenarios. A study based on consecutive human immunodeficiency virus screening conducted in Bihar, India, in 2014, found a coinfection rate of 5.6% (27). Human immunodeficiency virus increases the risk of developing leishmaniasis by up to 2,320 times, due to the virus’s opportunistic action, which compromises the immune system and facilitates the emergence of infections such as visceral leishmaniasis. Both diseases are defining conditions, as they promote negative mutualism in the cellular immune response, worsening the clinical condition of patients (28).

Regarding the confirmation criterion, laboratory tests were the most common, as was also the case in the states of Sergipe and Piauí (9,24). The reliability of laboratory diagnosis for visceral leishmaniasis is widely recognized and essential for effective disease management. According to the epidemiological report on leishmaniasis in the Americas, 88.0% of cases are diagnosed through laboratory testing, reinforcing the accuracy and reliability of these diagnostic methods (5). This data highlights the importance of early and accurate diagnosis, especially among vulnerable populations, such as pediatric patients, for whom appropriate treatment depends directly on laboratory confirmation of infection (9,24,28).

Regarding factors associated with death due to visceral leishmaniasis, living in the rural zone was found to be a risk factor for death in both the univariate (OR 2.19; 95%CI 1.10; 4.34) and the multivariate analysis (OR 2.96; 95%CI 1.35; 6.50). This finding may be attributed to late detection of the disease and delayed initiation of treatment in rural areas, where health infrastructure is more limited. In these regions, health facilities often lack the training for laboratory diagnosis of visceral leishmaniasis, reflecting a critical deficiency in diagnostic capacity, which aggravates the clinical picture and contributes to increased mortality (7,11).

Absence of weight loss was highlighted as a risk factor in the crude analysis (OR 1.68; 95%CI 1.02; 3.50), which may seem counterintuitive at a first glance, since weight loss is a characteristic symptom of this disease (9,16,28). However, this finding can be explained by the fact that patients who do not experience significant weight loss may have the disease diagnosed at more advanced stages, as this symptom often leads patients to seek medical help. Absence of weight loss can mask the severity of the disease, resulting in delayed treatment and, consequently, poorer prognosis.

Still regarding the analysis of associated factors, in the crude analysis, edema was identified as a risk factor (OR 1.93; 95%CI 0.99; 3.74), suggesting a tendency for greater severity in patients affected by this condition. Similarly, jaundice also stood out as a significant risk factor (OR 2.13; 95%CI 1.07; 4.24), which reinforces the need for special attention to these clinical signs for early intervention and reduction of mortality (16,20).

The 1 to 4 years age variable (OR 0.23; 95%CI 0.11; 0.50) proved to be a protective factor against death from visceral leishmaniasis. Individuals in this age group had lower odds of death compared to other age groups, suggesting that improvements in nutritional status and the inclusion of vaccinations, which strengthen the immune system, may have contributed to the reduction in mortality in this group (7,11). Other contributing factors include greater health care in early childhood, with increased pediatric follow-up, more frequent monitoring of growth and development, improved nutritional status, and early identification of signs of complications (13,19).

Although patients under 5 years of age are diagnosed earlier, they have a shorter survival time, probably due to their lower resistance to disease severity, despite timely diagnosis (29). Similarly, a study found that the 5-19 year age group had the lowest likelihood of death, which suggests that mortality in younger patients may vary according to different clinical and epidemiological contexts (30).

The findings of this research highlight the urgent need for targeted strategies to improve early diagnosis and management of visceral leishmaniasis, especially in rural areas where health infrastructure may be limited. The protection afforded to the 1-4 year age group suggests that interventions targeted at this population, including optimizing nutritional status and vaccination, can play a crucial role in reducing mortality due to visceral leishmaniasis. Continued efforts to understand and address the risk factors associated with mortality and promote improvements in diagnosis and treatment practices are essential for the effectiveness of visceral leishmaniasis control and prevention strategies.
