# Supplementary material for: Factors associated with deaths due to visceral leishmaniasis among children at a hospital in the southwest of Maranhão state: a cross-sectional study, Imperatriz, 2010-2021
Source: Epidemiol Serv Saude. 2025 Sep 29;34:e20240738. doi: 10.1590/S2237-96222025v34e20240738.en (PMC12479024; doi:10.1590/S2237-96222025v34e20240738.en)
Supplement: Translation

# Translation: Fatores associados a óbitos por leishmaniose visceral em crianças em um hospital do sudoeste maranhense: estudo transversal, Imperatriz, 2010-2021

**DOI:** 10.1590/S2237-96222025v34e20240738.pt

**Authors:** Marcela de Maria Pereira Teixeira, Daniel Ferreira dos Santos, Ezequiel Almeida Barros, Leonardo Hunaldo dos Santos, Laise Sousa Siqueira, Livia Maia Pascoal, Ana Cristina Pereira de Jesus Costa, Marcelino Santos

**Affiliations:** 1 Universidade Federal do Maranhão, Centro de Ciências de Imperatriz, Imperatriz, MA, Brasil

**Keywords:** Leishmaniose Visceral, Evolução Clínica, Criança, Vigilância em Saúde Pública, Análise Transversal

## Abstract

**Objetivos:**

Descrever as características clínico-epidemiológicas e analisar os fatores associados aos óbitos por leishmaniose visceral em crianças de um serviço hospitalar do sudoeste maranhense.

**Métodos:**

Tratou-se de estudo transversal, realizado em Imperatriz, Maranhão, a partir das fichas de notificação de agravos do Sistema de Informação de Agravos de Notificação disponibilizadas pelo Núcleo de Vigilância Epidemiológica e Controle de Doenças da unidade regional. Incluíram-se no estudo todos os registros de casos de leishmaniose visceral em crianças internadas em um hospital de referência pediátrica de Imperatriz entre 2010 e 2021. Os dados foram analisados com estatística descritiva, determinando valores absolutos e relativos das variáveis investigadas. Razões de chances (*odds ratio*, OR) e intervalos de confiança de 95% (IC95%) foram calculados por regressão uni e multivariada.

**Resultados:**

Foram registrados 404 casos do agravo, dos quais 43 evoluíram para óbito. Destes, a maioria era de casos novos (95,4%), sexo feminino (53,5%), raça/cor da pele parda (65,1%), residentes na zona urbana (60,5%) que apresentaram manifestações clínicas como aumento do baço (88,4%), palidez cutânea (86,0%), emagrecimento (69,8%), presença de edema (60,5%), febre (95,4%) e aumento do fígado (51,2%). No modelo final da regressão, residir na zona rural foi considerado fator de risco (OR 2,96; IC95% 1,35; 6,50), e a idade entre 1 e 4 anos apresentou-se como fator de proteção (OR 0,23; IC95% 0,11; 0,50) para o óbito pela doença.

**Conclusão:**

Os achados destacaram a necessidade de estratégias direcionadas para melhorar o diagnóstico precoce e o manejo de leishmaniose visceral, especialmente em áreas rurais.

**Aspectos éticos**

A pesquisa respeitou os princípios éticos, obtendo os seguintes dados de aprovação:

Comitê de ética em pesquisa: Universidade Federal do Maranhão

Número do parecer: 5.656.905

Data de aprovação: 21/9/2022

Certificado de apresentação de apreciação ética: 57683422.1.0000.5086

Registro do consentimento livre e esclarecido: Dispensado.

## Introdução

A leishmaniose visceral é uma doença tropical e crônica causada pelo protozoário *Leishmania chagasi*, pertencente à família *Trypanosomatidae* (1). No Brasil, a transmissão dessa doença ocorre através da picada da fêmea infectada do flebotomíneo *Lutzomyia longipalpis*, sendo o cão doméstico o principal reservatório. Com relação ao período de incubação da doença, este varia de 10 a 14 dias, e a sintomatologia pode aparecer entre dois e oito meses após a infecção. As manifestações clínicas incluem episódios irregulares de febre, perda de peso, fraqueza, aumento do baço e fígado, linfadenopatia e anemia (2). 

Dados globais de 2023 apontaram a leishmaniose visceral como uma doença endêmica em 83 países, e estima-se que surjam entre 50 mil e 90 mil casos novos a cada ano. Em 2018, mais de 90,0% dos casos novos ocorreram na Índia, em Bangladesh, no Brasil e no Leste Africano (3). 

Para a Organização Pan-Americana da Saúde, o continente americano enfrenta grandes desafios no que se refere ao controle da doença, devido à alta incidência e à ampla distribuição geográfica. No continente, o Brasil lidera em número de casos, registrando 7.928 entre 2018 e 2020, seguido por Venezuela, Paraguai, Colômbia, Argentina, Bolívia e Uruguai (4).

Em 2018, o Brasil concentrou cerca de 96,0% dos casos de leishmaniose visceral nas Américas, e o Nordeste foi a região mais afetada (5). Em 2020, dos 165 óbitos registrados no país, 107 ocorreram no Nordeste, e o Maranhão foi o estado que apresentou o maior número de mortes, com 42 dos 165 casos entre as Unidades da Federação (6-8).

A leishmaniose visceral possui alta letalidade quando não tratada. Cerca de 90,0% dos casos podem evoluir ao óbito, por consequência do envolvimento sistêmico causado pela presença de parasitas em órgãos internos como medula óssea, baço e fígado. A doença afeta principalmente populações mais vulneráveis, como crianças menores de 5 anos de idade, idosos, pacientes com comorbidades e outras condições com imunossupressão, a exemplo de infecção pelo vírus da imunodeficiência humana/síndrome da imunodeficiência adquirida e desnutrição.

Em 2020, de 162 casos notificados de óbitos por leishmaniose visceral, 16,0% eram crianças menores de 5 anos de idade (4). Nesse mesmo ano, a taxa de letalidade pela doença atingiu 9,5%, a mais alta dos últimos 10 anos, com destaque para o preocupante índice entre crianças menores de 1 ano de idade, que chegou a 14,0% (8). A população infantil é a mais afetada pela leishmaniose visceral (9-12).

A compreensão aprofundada da epidemiologia e da evolução da leishmaniose visceral em populações infantis é essencial para o desenvolvimento de estratégias eficazes de prevenção e controle. Apesar dos avanços nas investigações científicas ao longo dos anos, ainda são desconhecidos os fatores específicos que contribuem para os óbitos por leishmaniose visceral em crianças. Supõe-se que a vulnerabilidade imunológica desses indivíduos, aliada à maior propensão à desnutrição, possa agravar o quadro clínico da doença, o que aumenta o risco de desfechos fatais (7,13). Diante dessa lacuna de conhecimento, torna-se imperativa a realização de estudos que ampliem a compreensão epidemiológica da leishmaniose visceral em diferentes cenários endêmicos.

Este estudo objetivou descrever as características clínico-epidemiológicas e analisar os fatores associados aos óbitos por leishmaniose visceral em crianças de um serviço hospitalar de Imperatriz.

## Métodos

### Delineamento 

Tratou-se de estudo transversal que investigou fatores clínico-epidemiológicos associados ao óbito por leishmaniose visceral em crianças entre 2010 e 2021 de um serviço hospitalar de Imperatriz.

### Contexto

Os dados do estudo foram oriundos das fichas de notificação de agravos do Sistema de Informação de Agravos de Notificação disponibilizadas pelo Núcleo de Vigilância Epidemiológica e Controle de Doenças da Unidade Regional de Saúde de Imperatriz. Consideraram-se os registros de leishmaniose visceral na faixa etária de 0-12 anos de um hospital do sudoeste maranhense, situado em Imperatriz. 

Esse município é a segunda maior cidade do Maranhão e integra a macrorregião sul de saúde, junto com municípios como Balsas, Açailândia e Barra do Corda. O Hospital Municipal Infantil de Imperatriz é a única unidade pública de referência em pediatria da região e oferece serviços de urgência e emergência, cirurgias, atendimento clínico pediátrico e unidade de terapia intensiva.

### Participantes

Foram incluídos no estudo todos os casos de óbitos por leishmaniose visceral registrados em crianças menores de 12 anos de idade internadas na unidade hospitalar entre 2010 e 2021. No entanto, ressalta-se que, apesar do fator de inclusão citado, somente foram encontrados óbitos em crianças de 0 a 4 anos de idade.

### Variáveis

As variáveis analisadas incluíram tipo de entrada, sexo, faixa etária, raça/cor da pele, zona de residência, manifestações clínicas (como aumento do baço, palidez, emagrecimento, edema, febre, icterícia e aumento do fígado), coinfecção por vírus da imunodeficiência humana, métodos diagnósticos e critérios de confirmação diagnóstica.

### Fontes de dados e mensuração 

A coleta de dados foi realizada por meio das fichas de notificação de agravos do Sistema de Informação de Agravos de Notificação, referentes à leishmaniose visceral. Esses dados foram disponibilizados pelo Núcleo de Vigilância Epidemiológica e Controle de Doenças da Unidade Regional de Saúde de Imperatriz, vinculado à Secretaria de Estado da Saúde do Maranhão. As variáveis foram devidamente codificadas e registradas de acordo com os padrões do sistema, assegurando a uniformidade na mensuração dos dados.

### Controle de viés

Foram adotadas medidas para minimizar viés, incluindo a exclusão de dados ignorados ou incompletos e a verificação de multicolinearidade entre variáveis, assegurando que a análise estatística refletisse a realidade dos casos avaliados.

### Tamanho da amostra

A análise incluiu todos os registros disponíveis de casos de óbitos por leishmaniose visceral em crianças menores de 12 anos de idade no período 2010-2021. A amostra compreendeu 43 casos de óbitos por leishmaniose visceral no período investigado.

### Avaliação da qualidade dos dados

Os dados coletados foram revisados para identificar e corrigir possíveis inconsistências ou erros. Dados incompletos ou ignorados foram excluídos para garantir a precisão da análise. Não houve multicolinearidade entre as variáveis independentes avaliadas. Nesta ocasião, os dados ignorados foram excluídos. Todos os testes foram realizados no software Statistical Package for the Social Sciences (IBM SPSS) Statistics com 5% de significância (15).

### Métodos estatísticos

A análise inicial foi realizada por meio de regressão logística univariada (não ajustada) para verificar associações entre as variáveis e o desfecho óbito por leishmaniose visceral, considerando p-valor<0,20 para a seleção. As variáveis significativas foram posteriormente analisadas em uma regressão logística multivariada (ajustada) para estimar as razões de chances (*odds ratio*, OR) com intervalos de confiança de 95% (IC95%), mantendo o nível de significância de 5% (14). 

## Resultados

No período compreendido entre 2010 e 2021, foram confirmados 404 casos de leishmaniose visceral em menores de 12 anos de idade, dos quais 43 (7,7%) evoluíram para óbito. A maioria dos casos de óbito era caso novo (41; 95,4%), sexo feminino (23; 53,5%), raça/cor da pele parda (28; 65,1%) e residentes na zona urbana (26; 60,5%). Entre as principais manifestações clínicas, destacaram-se aumento do baço (38; 88,4%), palidez (37; 86,0%), emagrecimento (30; 69,8%), presença de edema (26; 60,5%), febre (41; 95,4%), sem icterícia (24; 55,8%) e com aumento do fígado (22; 51,2%). Destacou-se a não ocorrência da coinfecção leishmaniose visceral/vírus da imunodeficiência humana (37; 86,0%) entre os casos de óbitos notificados (Tabela 1).

**Tabela 1:** Distribuição do número de casos (n) e porcentagens (%) de acordo com as características clínico-epidemiológicas e laboratoriais dos óbitos por leishmaniose visceral em crianças internadas em serviço de pediatria no sudoeste maranhense entre 2010 e 2021. Imperatriz, 2025 (n=43)

Variáveis	n (%)
**Tipo de entrada**	
Caso novo	41 (95,4)
Recidiva	1 (2,3)
Ignorado	1 (2,3)
Sexo	
Masculino	20 (46,5)
Feminino	23 (53,5)
**Faixa etária** (anos)	
<1	20 (46,5)
1-4	21 (48,8)
Ignorado	2 (4,7)
**Raça/cor da pele**	
Branca	6 (14,0)
Parda	28 (65,1)
Indígena	7 (16,2)
Ignorado	2 (4,7)
**Zona de residência**	
Urbana	26 (60,5)
Rural	16 (37,2)
Periurbana	1 (2,3)
**Aumento do baço**	
Sim	38 (88,4)
Não	3 (6,9)
Ignorado	2 (4,7)
Palidez	
Sim	37 (86,0)
Não	5 (11,7)
Ignorado	1 (2,3)
Emagrecimento	
Sim	30 (69,8)
Não	12 (27,9)
Ignorado	1 (2,3)
Edema	
Sim	26 (60,5)
Não	16 (37,2)
Ignorado	1 (2,3)
Febre	
Sim	41 (95,4)
Não	1 (2,3)
Ignorado	1 (2,3)
Icterícia	
Paciente ictérico	16 (37,2)
Paciente anictérico	24 (55,8)
Ignorado	3 (7,0)
**Aumento do fígado**	
Sim	22 (51,2)
Não	18 (41,8)
Ignorado	3 (7,0)
**Vírus da imunodeficiência humana**	
Sim	1 (2,3)
Não	37 (86,0)
Ignorado	5 (11,7)
**Diagnóstico parasitológico**	
Positivo	7 (16,3)
Negativo	1 (2,3)
Ignorado	35 (81,4)
**Diagnóstico imunológico**	
Positivo	12 (27,9)
Negativo	7 (16,3)
Ignorado	24 (55,8)
**Critério de confirmação**	
Laboratorial	25 (58,1)
Clínico epidemiológico	18 (41,9)

Em relação ao critério de confirmação, o laboratorial (25; 58,1%) foi o mais evidente. No que se refere ao diagnóstico da doença, as variáveis diagnóstico parasitológico (35; 81,4%) e diagnóstico imunológico (24; 55,8%) apresentaram inconsistência dos dados e consequentemente maior frequência de dados ignorados (Tabela 1).

A análise bruta das variáveis que apresentaram associação significativa com o óbito por leishmaniose visceral identificou como fatores de risco: residir na zona rural (OR 2,19; IC95% 1,10; 4,34), o não emagrecimento (OR 1,68; IC95% 1,02; 3,50), edema (OR 1,93; IC95% 1,09; 3,74), icterícia (OR 2,13; IC95% 1,07; 4,24) e critério de confirmação clínico epidemiológico (OR 1,62; IC95% 1,09; 3,13) (Tabela 2). A variável idade de 1 a 4 anos (OR 0,29; IC95% 0,14; 0,56) mostrou-se como fator de proteção por ter apresentado p-valor<0,001.

**Tabela 2:** Razões de chances (*odds ratio*, OR) brutas e ajustadas e intervalo de confiança de 95% (IC95%) dos óbitos por leishmaniose visceral em crianças internadas em serviço de pediatria no sudoeste maranhense entre 2010 e 2021 pelas variáveis do estudo. Imperatriz, 2025 (n=404)

Variáveis	OR bruta (IC95%)	p-valor	OR ajustada (IC95%)	p-valor
**Tipo de entrada**		0,380		-
Caso novo	1,00			
Recidiva	0,40 (0,52; 3,09)		-	
Sexo		0,300		-
Masculino	1,00			
Feminino	1,40 (0,74; 2,67)		-	
**Faixa etária** (anos)		<0,001		<0,001
<1	1,00			
1-4	0,29 (0,14; 0,56)		0,23 (0,11; 0,50)	
**Raça/cor da pele**		0,570		-
Parda	1,26 (0,57; 2,79)		-	
Não parda	1,00			
**Zona de residência**		0,030		0,010
Urbana	1,00			
Rural	2,19 (1,10; 4,34)		2,96 (1,35; 6,50)	
Periurbana			0,00	1
**Aumento do baço**		0,630		-
Sim	1,36 (0,39; 4,69)		-	
Não	1,00			
Palidez		0,700		-
Sim	1,00			
Não	1,22 (0,44; 3,36)		-	
Emagrecimento		0,160		0,290
Sim	1,00			
Não	1,68 (1,02; 3,50)		0,64 (0,28; 1,48)	
Edema		0,050		0,460
Sim	1,93 (1,09; 3,74)		1,33 (0,63; 2,86)	
Não	1,00			
Febre		0,780		-
Sim	1,35 (0,17; 10,95)		-	
Não	1,00			
Icterícia		0,030		0,180
Sim	2,13 (1,07; 4,24)		1,73 (0,78; 3,80)	
Não	1,00			
**Aumento do fígado**		0,990		-
Sim	1,00			
Não	1,05 (0,52; 1,96)		-	
**Vírus da imunodeficiência humana**		0,990		-
Sim	1,00			
Não	1,02 (0,12; 8,50)		-	
**Diagnóstico parasitológico**		0,910		-
Positivo	0,14 (0,12; 10,94)		-	
Negativo	1,00			
**Diagnóstico imunológico**		0,520		-
Positivo	1,00			
Negativo	1,39 (0,51; 3,77)		-	
**Critério de confirmação**		0,150		0,200
Laboratorial	1,00			
Clínico epidemiológico	1,62 (1,09; 3,13)		1,65 (0,76; 3,60)	

Na análise ajustada, a variável que se apresentou como fator de risco para óbito por leishmaniose visceral foi residir na zona rural (OR 2,96; IC95% 1,35; 6,50).

A variável idade entre 1 e 4 anos (OR 0,23; IC95% 0,11; 0,50) apresentou-se como fator de proteção (Tabela 2).

## Discussão

Foram registrados 404 casos de leishmaniose visceral na faixa etária indicada, dos quais 43 evoluíram para óbito. Destes, a maioria era de casos novos, sexo feminino, raça/cor da pele parda, residentes na zona urbana que apresentaram manifestações clínicas como aumento do baço, palidez cutânea, emagrecimento, presença de edema, febre e aumento do fígado. No modelo final da regressão, residir na zona rural foi considerada fator de risco, e a idade entre 1 e 4 anos apresentou-se como fator de proteção para o óbito pela doença.

Cabe ressaltar a limitação do estudo decorrente do número de dados ignorados, o que evidencia variações na qualidade do preenchimento das informações e restringe as variáveis que poderiam ser exploradas. Essa limitação pode gerar vieses na interpretação dos dados e até mesmo indicar subnotificações. Assim, destaca-se a importância de melhorar a qualidade dos registros, especialmente no preenchimento das notificações, uma vez que essas informações são fundamentais para o planejamento e a gestão eficiente em saúde. Outro fator a ser destacado é que, apesar de serem analisados óbitos na faixa etária de 0-12 anos, somente foram encontrados óbitos em crianças de até 4 anos de idade.

Analisando as características clínico-epidemiológicas, o estudou apontou que a variável caso novo apresentou alto percentual referente ao tipo de entrada de óbito pelo agravo. Em Mato Grosso, foi observado que a faixa etária de 0-4 anos teve 24,2% dos óbitos, com a taxa de 96,2% entre os casos novos em 2022 (16). No Pará, as crianças constituíram a população particularmente susceptível, e, em 2021, houve o maior percentual de óbitos (83,3%) entre os casos de infecção primária (13).

Observou-se, ainda, que pacientes do sexo feminino obtiveram maior frequência relativa de óbitos em relação ao sexo masculino. Houve divergência em relação a cenários brasileiros, como no Tocantins, em Pará, em Sergipe, em Mato Grosso e no Piauí, onde o sexo masculino foi o mais acometido (9,17-20). O fato de o sexo masculino ser o mais acometido em relação ao adoecimento e óbito ainda não está totalmente esclarecido.

Devido à escassez de estudos que apontam tal evidência, uma hipótese atribuída são as mesmas causas fisiológicas atribuídas aos homens na fase adulta, relacionadas aos efeitos imunológicos dos hormônios sexuais, tornando-os mais susceptíveis à Leishmania, somado ao fato da primeira infância ser a fase em que o indivíduo tem o sistema imunológico imaturo (21-23).

Os óbitos por leishmaniose visceral ocorreram com maior proporção em indivíduos da raça/cor da pele parda, em consonância com dados encontrados no Piauí em 2022, em que 100,0% dos casos de óbitos foram em pacientes pardos (24). Esse expressivo percentual pode ser explicado pelo fato de a leishmaniose visceral ser uma doença negligenciada, que afeta predominantemente indivíduos em condições socioeconômicas desfavoráveis, contexto em que se encontra a maioria da população não branca (20,23).

A maior ocorrência de óbitos se deu em indivíduos residentes da zona urbana, assim como foi constatado no Pará em 2021 (19). Sugere-se que esses achados estejam relacionados a fatores decorrentes das limitações enfrentadas pelos serviços de saúde locais, bem como ao desconhecimento no manejo adequado de pacientes com leishmaniose visceral. Isso inclui a identificação tardia da necessidade de encaminhamento de casos graves para centros de referência com maior capacidade tecnológica, o que poderia contribuir para a redução da letalidade da doença (25).

Os resultados obtidos revelaram a predominância de manifestações clínicas clássicas da leishmaniose visceral entre os pacientes pediátricos, reforçando o caráter sistêmico e grave da doença. O aumento do baço e do fígado foram achados comuns, alinhados à patogênese da leishmaniose visceral, que frequentemente provoca esplenomegalia e hepatomegalia, devido à invasão parasitária dos órgãos linfoides e hepáticos. A febre e a palidez destacaram-se como marcadores importantes de infecção e anemia, respectivamente, corroborando a literatura que associa tais sintomas à desnutrição e ao comprometimento imunológico dos pacientes (9,20,26).

A alta taxa de emagrecimento e edema sugere, ainda, o impacto nutricional severo e o comprometimento metabólico gerado pela doença, agravado pelas condições socioeconômicas desfavoráveis, que contribuem para o quadro clínico debilitante dessas crianças. Esses achados sublinham a necessidade urgente de intervenções precoces para evitar complicações fatais e melhorar o manejo clínico de casos pediátricos (9,20,26).

Este estudo destacou a não ocorrência da coinfecção leishmaniose visceral/vírus da imunodeficiência humana, um achado que contrasta com outros cenários globais. Em uma triagem consecutiva de vírus da imunodeficiência humana realizada em Bihar, na Índia, em 2014, a taxa de coinfecção foi 5,6% (27). O vírus da imunodeficiência humana eleva o risco de desenvolvimento da leishmaniose em até 2.320 vezes, devido à ação oportunista do vírus, que compromete o sistema imunológico e facilita o surgimento de infecções como a leishmaniose visceral. Ambas as doenças são condições definidoras, pois promovem mutualismo negativo na resposta imune celular, agravando o quadro clínico dos pacientes (28).

Com relação ao critério de confirmação, o laboratorial ocorreu em maior proporção, assim como foi encontrado em Sergipe e no Piauí (9,24). A confiabilidade do diagnóstico laboratorial para leishmaniose visceral é amplamente reconhecida e essencial para o manejo eficaz da doença. De acordo com o informe epidemiológico de leishmanioses nas Américas, 88,0% dos casos são diagnosticados por meio de testes laboratoriais, reforçando a precisão e a confiança nesses métodos diagnósticos (5). Esse dado evidencia a importância de diagnóstico precoce e acurado, especialmente em populações vulneráveis, como pacientes pediátricos, em que o tratamento adequado depende diretamente da confirmação laboratorial da infecção (9,24,28).

Referente aos fatores associados ao óbito por leishmaniose visceral, observou-se que residir na zona rural se comportou como fator de risco para óbito tanta na análise univariada (OR 2,19; IC95% 1,10; 4,34), como na multivariada (OR 2,96; IC95% 1,35; 6,50). Esse achado pode ser atribuído à detecção tardia da doença e ao atraso no início do tratamento em áreas rurais, onde a infraestrutura de saúde é mais limitada. Nessas regiões, as unidades de saúde frequentemente carecem de capacitação para o diagnóstico laboratorial de leishmaniose visceral, refletindo a deficiência crítica na capacidade diagnóstica, o que agrava o quadro clínico e contribui para o aumento da letalidade (7,11).

O não emagrecimento foi destacado como fator de risco na análise bruta (OR 1,68; IC95% 1,02; 3,50), o que pode parecer contraintuitivo à primeira vista, já que a perda de peso é um sintoma característico dessa patologia (9,16,28). No entanto, esse achado pode ser explicado pelo fato de que pacientes que não apresentam emagrecimento significativo podem ter a doença diagnosticada em estágios mais avançados, visto que esse sintoma costuma levar os pacientes a procurarem ajuda médica. O não emagrecimento pode mascarar a gravidade da doença, resultando em atraso no tratamento adequado e, consequentemente, em pior prognóstico.

Ainda na análise de fatores associados, na análise bruta, o edema foi identificado como fator de risco (OR 1,93; IC95% 0,99; 3,74), sugerindo uma tendência de maior gravidade nos pacientes acometidos por essa condição. Da mesma forma, a icterícia também se destacou como fator de risco significativo (OR 2,13; IC95% 1,07; 4,^24^), o que reforça a necessidade de atenção especial a esses sinais clínicos para uma intervenção precoce e redução da letalidade (16,20).

A variável idade entre 1 e 4 anos (OR 0,23; IC95% 0,11; 0,50) mostrou-se como fator de proteção contra o óbito por leishmaniose visceral. Indivíduos nessa faixa etária apresentaram menor chance de óbito em comparação com outras idades, o que sugeriu que melhorias no estado nutricional e a inclusão de vacinas, que fortalecem o sistema imunológico, podem ter contribuído para a redução da mortalidade nesse grupo (7,11). Outros fatores que contribuem são a maior atenção à saúde na primeira infância, com o aumento do acompanhamento pediátrico, o monitoramento mais frequente do crescimento e desenvolvimento, além de condições nutricionais aprimoradas e a identificação precoce de sinais de complicações (13,19).

Embora pacientes menores de 5 anos de idade sejam diagnosticados mais cedo, estes apresentam menor tempo de sobrevida, provavelmente devido à sua menor resistência à gravidade da doença, apesar do diagnóstico oportuno (29). Da mesma forma, a faixa etária de 5-19 anos apresentou as menores chances de óbito, o que sugere que a mortalidade em pacientes mais jovens pode variar de acordo com diferentes contextos clínicos e epidemiológicos (30).

Os achados desta pesquisa destacam a necessidade urgente de estratégias direcionadas para melhorar o diagnóstico precoce e o manejo de leishmaniose visceral, especialmente em áreas rurais, onde a infraestrutura de saúde pode ser limitada. A proteção conferida à faixa etária de 1-4 anos sugere que intervenções voltadas para essa população, incluindo a otimização das condições nutricionais e vacinação, podem desempenhar papel crucial na redução da mortalidade por leishmaniose visceral. A continuidade de esforços para entender e abordar os fatores de risco associados à mortalidade e promover melhorias nas práticas de diagnóstico e tratamento é essencial para a eficácia das estratégias de controle e prevenção da leishmaniose visceral.
